# Supplementary material for: Heterozygote Dopamine Transporter Knockout Rats Display Enhanced Cocaine Locomotion in Adolescent Females
Source: Int J Mol Sci. 2022 Dec 6;23(23):15414. doi: 10.3390/ijms232315414 (PMC9736933; doi:10.3390/ijms232315414)
Supplement: Supplementary file 1 [file ijms-23-15414-s001.zip › ijms-2044131-supplementary.pdf]

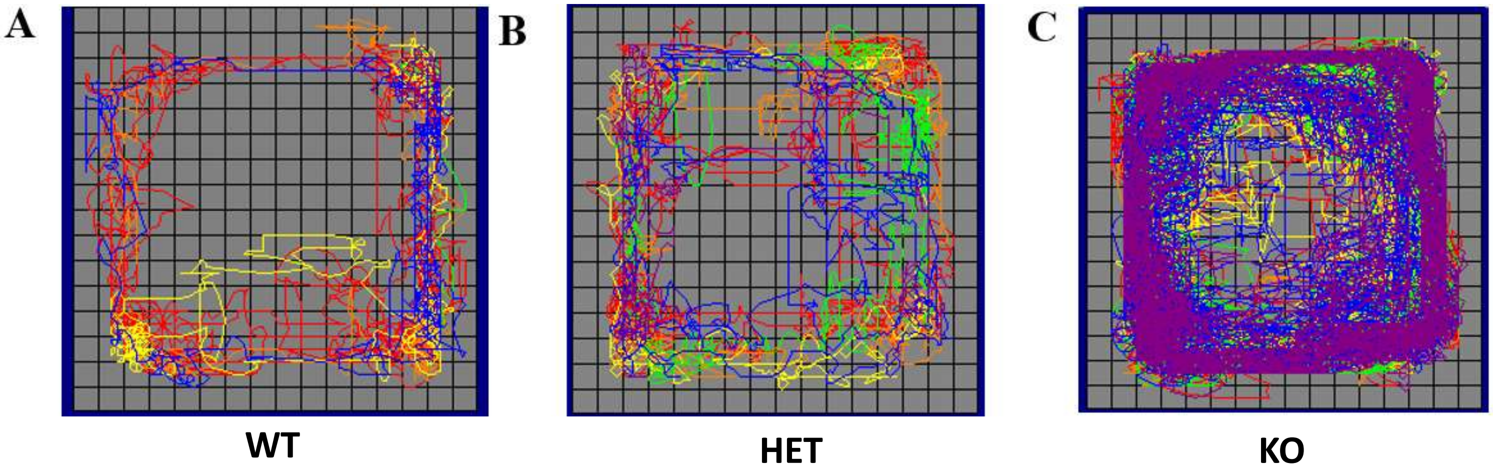

Supplementary Figure S1

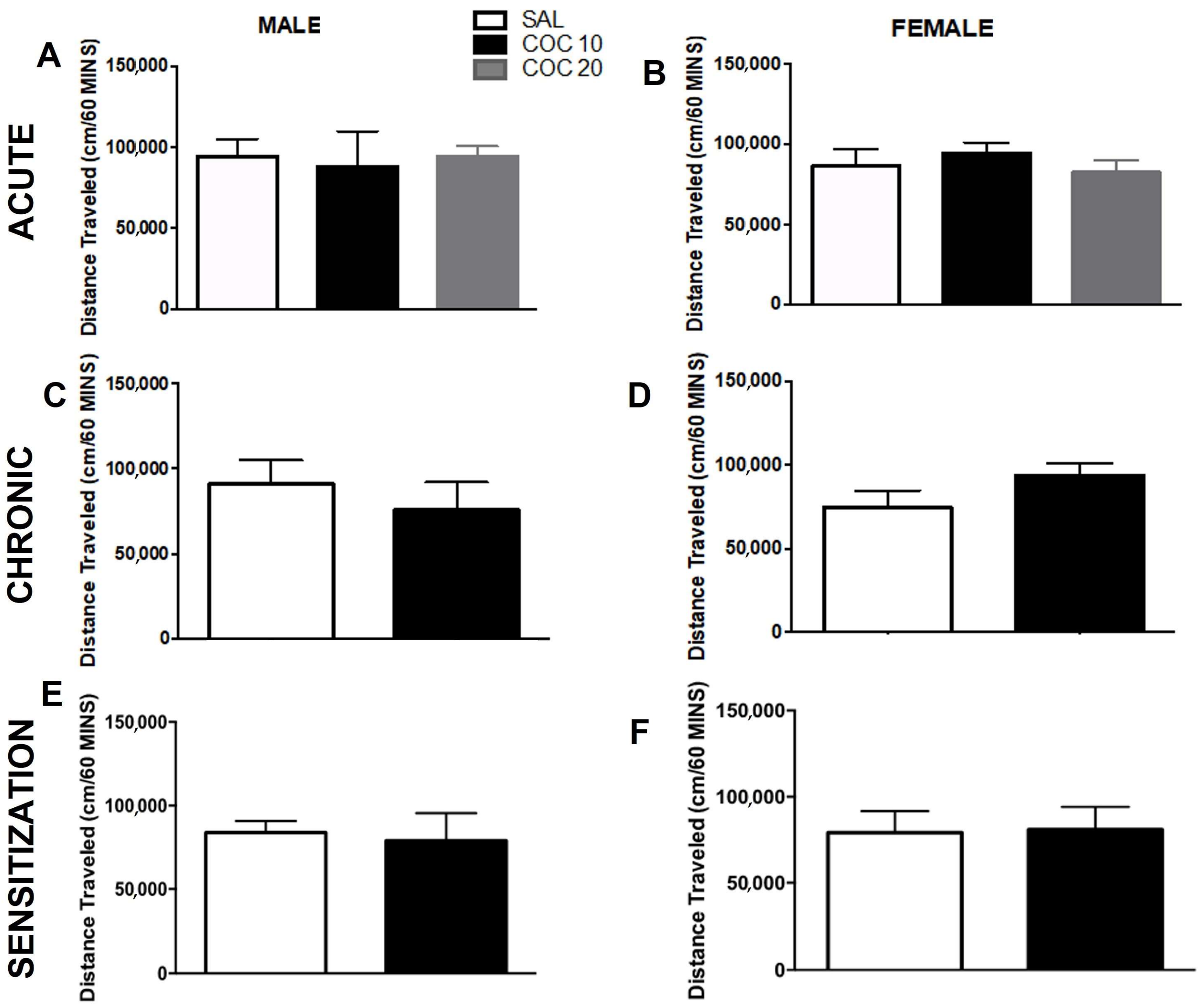

Supplementary Figure S2

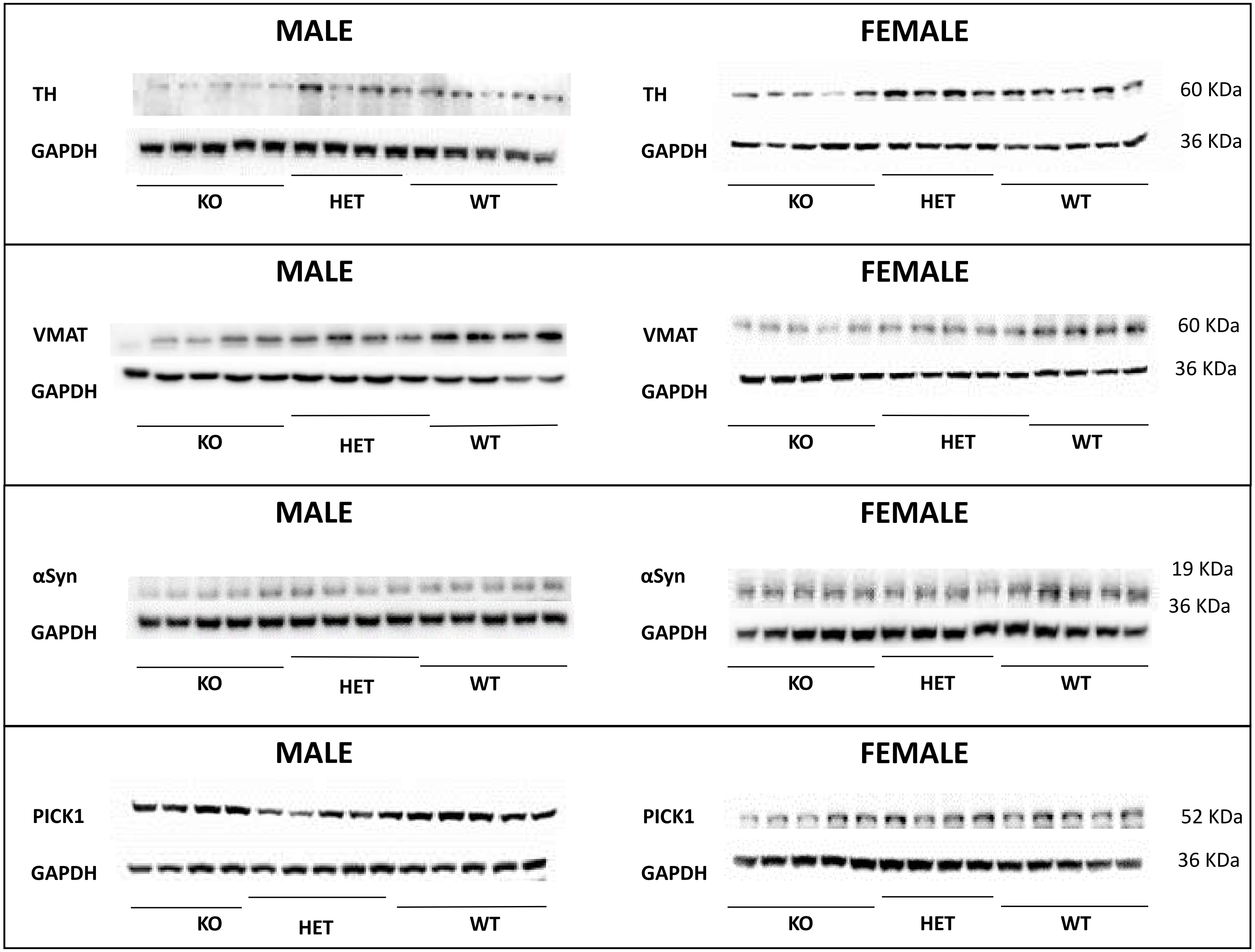

Supplementary Figure S3

Supplementary Figure S1. Illustrative example of Wild-type (WT) (A), Heterozygous (HET) (B) and Knockout (KO) (C) rat's travel pathway on the open field during 30 minutes of habituation.

Supplementary Figure S2. Acute, chronic and sensitization effects of cocaine on locomotor activity in KO DAT rats. Cumulative time (30 min after acute treatment) is represented across conditions (SAL=saline, COC 10= 10mg/kg cocaine; COC 20= 20mg/kg cocaine) in males (A,C,D) and females (B,D,F), during acute (A,B), chronic (C,D) and sensitization (E,F) conditions. Mean ± SEM. (males: KO N=17; females: KO N=23).

Supplementary Figure S3. Western blot images of striatal protein expression of A) Tyrosine-hydroxylase (TH), B) Vesicular monoamine transporter 2 (VMAT2), C) α-Synuclein (α-Syn) and D) protein interacting with C-kinase-1 (PICK) in DAT mutant rats
